# Female genital mutilation/cutting in Sierra Leone: are educated women intending to circumcise their daughters?

**DOI:** 10.1186/s12914-020-00240-0

**Published:** 2020-07-23

**Authors:** Edward Kwabena Ameyaw, Justice Kanor Tetteh, Ebenezer Kwesi Armah-Ansah, Kofi Aduo-Adjei, Aisha Sena-Iddrisu

**Affiliations:** 1 The Australian Centre for Public and Population Health Research, Faculty of Health, University of Technology Sydney, Sydney, NSW Australia; 2 Department of Population and Health, College of Humanities and Legal Studies, University of Cape Coast, Cape Coast, Ghana; 3 Centre for Health Services Management, Faculty of Health, University of Technology Sydney, Sydney, NSW Australia; 4 Nursing and Midwifery Training College, Sunyani, Ghana

**Keywords:** Female genital mutilation, Female genital cutting, Circumcision, Education, Sierra Leone

## Abstract

**Background:**

Female genital mutilation/cutting (FGM/C) has been recognized as a gross violation of human rights of girls and women. This is well established in numerous international legal instruments. It forms part of the initiation ceremony that confers womanhood in Sierra Leone. Girls and women who are subjected to this practice are considered to be ready for marriage by their parents and communities and are rewarded with celebrations, gifts, and public recognition. Following this, we examined the relationship between education and women’s FGM/C intention for their daughters in Sierra Leone.

**Methods:**

We used cross-sectional data from the women’s file of the 2013 Sierra Leone Demographic and Health Survey (SLDHS) to explore the influence of education on FGM/C intention among women in the reproductive age (15–49). A sample of 6543 women were included in the study. Our analysis involved descriptive computation of education and FGM/C intention. This was followed by a two-level multilevel analysis. Fixed effect results were reported as Odds Ratios and Adjusted Odds Ratios with their respective credible intervals (CrIs) whilst results of the random effects were presented as variance partition coefficients and median odds ratios.

**Results:**

Our findings showed that women who had no formal education were more likely to intend to circumcise their daughters [aOR = 4.3, CrI = 2.4–8.0]. Among the covariates, women aged 20–24 [aOR = 2.3, CrI = 1.5–3.4] were more likely to intend to circumcise their daughters compared to women between 45 and 49 years old. Poorest women were more likely to report intention of circumcising their daughters in the future compared with the richest [aOR = 2.1, CrI = 1.3–3.2]. We noted that, 63.3% of FGM/C intention in Sierra Leone is attributable to contextual factors.

**Conclusion:**

FGM/C intention is more common among women with no education, younger women as well as women in the lowest wealth category. We recommend segmented female-child educational and pro-poor policies that target uneducated women in Sierra Leone. The study further suggests that interventions to end FGM/C need to focus on broader contextual and social norms in Sierra Leone.

## Background

The fifth Sustainable Development Goal seeks to abolish all harmful practices including female genital mutilation/cutting (FGM/C) by the year 2030 [1]. FGM/C is a gross violation of human rights [2]. The 2018 World Bank’s Compendium of International and National Legal Frameworks on FGM/C indicate that about 60 countries have adopted anti FGM/C laws [3]. FGM/C is a harmful cultural and traditional practice in Sierra Leone, other countries in sub-Saharan African, Asia, Middle East and some communities in South America [2, 4, 5]. It has also been reported in some western countries such as Australia, New Zealand, Canada, and the United Kingdom [5]. FGM/C refers to all procedures involving total or partial removal of the external parts of female genitalia or any other injury to the genital organ [6, 7]. The WHO has identified four main types of FGM/C. This is based on the severity of injury to the genitalia and may not involve the removal of flesh, such as pricking, piercing or scraping (type 4), to infibulation, in which the vaginal opening is narrowed by cutting and repositioning the labia to create a partial cover (type 3) [8]. Type 1 involves the partial removal of the clitoral glans and/or clitoral hood whilst type 2 constitutes the partial or complete elimination of clitoral glans and labia minora with/without eliminating the labia majora [7, 9]. FGM/C is usually carried out on young females between infancy and 15 years [8]. FGM/C threatens the health and well-being of millions of girls, women and their children globally [1].

More than 200 million girls and women alive today have been cut in Africa, the Middle East and Asia [10]. In Africa, an estimated 92 million girls 10 years and older have undergone the practice [8]. Victims may die during the procedure due to haemorrhage or septic shock [11], or experience considerable physical, psychological and sexual complications [12–15]. Surveys conducted across Africa, the Middle East, and Southeast Asia document FGM/C prevalence rates ranging from 1% (Uganda, Cameroon) to more than 95% (Guinea and Somalia) [16]. Although some sub-Saharan African (SSA) communities believe that the procedure ensures virginity before marriage and keeps one’s sexual organs clean [17], researchers in Kenya and Nigeria observed no association between FGM/C and outcomes for sexual behaviour [18]. Social pressure has been cited as one of the principal reasons for FGM/C [7, 19, 20]. Others also consider it as removing “unclean” parts of the girl’s body which is essential for maintaining femininity [21] and enhancing marriage prospects [22, 23]. However, no scientific evidence support its medical benefits [24].

In the last decade, criminalization of FGM/C is gaining momentum in law including penal codes, and domestic violence acts [3]. Of the 29 FGM/C prevalent countries in Africa, 24 governments have enacted laws against the practice [25]. For example, countries such as Ghana, Burkina Faso, Ivory Coast, Senegal, Djibouti, and Togo have banned FGM/C [2]. In line with this, a number of professional associations including International Federation of Obstetricians and Gynaecologists as well as the International Confederation of Midwives denounce the practice and call on all professional associations to condemn it [25].

Consistent negative association has been reported between women’s education, FGM/C intention and its perpetration [26–30]. The common pathways for the negative association include the fact that education enlightens women to appreciate the consequences of FGM/C on females’ sexual and reproductive health [28] including mental health implications [31]. Education in effect empowers women to oppose all forms of violence against women and protect their children from experiencing FGM/C and abuse of all forms [30, 32]. Meanwhile, Hayford et al. [33] observed that daughters of women with primary or higher education experienced FGM/C in Burkina Faso, Cote d’voire, Guinea and Mali.

In Sierra Leone, FGM/C forms part of an initiation ceremony which confers womanhood, locally termed as ‘Bondo Bush.’ The ceremony is conducted secretly to usher girls into womanhood [34].

Girls and women who are subjected to FGM/C are perceived to be well trained, ready for marriage and are rewarded with celebrations, gifts, and public recognition in Sierra Leone [35]. It is believed that the physical pain endured helps to create a life-long fellowship among girls who are jointly initiated [34]. Sierra Leone is a member of several international human rights conventions which recognise FGM/C as a violation of human rights [36]. However, there is no prohibition of FGM/C, or any explicit law against the practice in Sierra Leone [29, 36]. Although the 2007 Child Rights Act supersedes all other national laws related to children’s rights, the FGM/C clause was removed from the final version during parliamentary debate [37].

In 2012, eight of the country’s fourteen districts signed a Memorandum of Understanding (MoU) criminalizing FGM/C among children (in Western Area Rural, Western Area Urban, Bo, Kambia, Port Loko, Pujehun, Bonthe, and Kailahun) but the practice continues in many parts of these districts without any consequences for offenders [2, 36]. The national statistics of Sierra Leone estimates that the prevalence of FGM/C slightly decreased from 91.3% in 2008 to 89.6% in 2013 [38]. The prevalence between females whose mothers have no education (95%) and females whose mothers have at least secondary education (74.2%) is higher in Sierra Leone than in neighbouring countries [34]. Sierra Leone is classified as a group one country with a prevalence of more than 80%, according to the UNICEF classification [36].

Several studies have examined factors that influence FGM/C. Some have highlighted the decision makers for the practice [34], the forms and health complications [6]. Yet, little is known about the relationship between education and intention to circumcise daughters in Sierra Leone. This study, therefore, seeks to investigate the linkage between education and intention to circumcise one’s daughter in the future. Findings and recommendations from the study will shape FGM/C policy formulation and evaluation of how best education can be utilised as a critical tool to end FGM/C in Sierra Leone and other FGM/C prevalent countries.

## Methods

### Data source

Data for this study was obtained from the 2013 Sierra Leone Demographic and Health Survey (SLDHS) [38]. The 2013 SLDHS happens to be the second version of the Demographic and Health Survey following the first one in 2008. The survey intended to gather data for monitoring the population and health circumstances and to serve as a follow-up to the first survey. It captured information on female genital mutilation, fertility preferences, maternal and child health as well as sexual activity. It was implemented by the Statistics Sierra Leone (SSL) under the auspices of the Ministry of Health and Sanitation. The National Technical Committee and National Steering Committee provided technical and policy guidance for the conduct of the survey. Inner City Fund (ICF) International also offered technical assistance via the MEASURE DHS Program [38].

Sample for the survey was designed to generate dependable estimates for essential variables for the entire country in both rural and urban locations as well as across the four regions and the fourteen districts [38]. To ensure representation for all the aforementioned demarcations, the sample was stratified and selected in two stages. Primary Sampling Units (PSUs), also known as clusters were selected based on the list of enumeration areas (EAs) created in the preceding Population and Housing Census in the first stage [38]. The EAs offered the principal frame for selecting 435 clusters (158 from urban and 277 from rural) through probability proportional to their sizes. Excluded from the sampling frame were persons residing in collective housing units including hospitals, hotels and boarding schools [38]. Thirty (30) households were systematically selected from each of the clusters in the second stage. All eligible persons in the subsample of households participated in the study. This comprised women aged 15–49 and men aged 15–59. Although, 17, 1323 eligible women (15–49 years) were identified in all, complete interviews were conducted with 16,658 and this culminated in a 97% response rate. Nearly half of these women had at least one daughter (46.7%). The three questionnaires used (household questionnaire, woman’s questionnaire, and man’s questionnaire) emerged from the models developed by the MEASURE DHS Program. However, the questionnaires were modified to fit the context of Sierra Leone [38].

### Study variables

#### Dependent variable

Intention to circumcise daughter in the future was the dependent variable for the study. As part of the survey, the women were asked “Do you intend to have daughter(s) circumcised in future?” The corresponding responses were ‘No’ coded as 0, ‘Yes’ coded as 1, and ‘Don’t Know’, coded as 8. To arrive at findings that reflect precision in thought and offer meaningful recommendations, women who responded ‘Don’t Know’ were excluded from the study.

#### Independent variable

The principal independent variable was formal education. In the DHS survey, formal education is categorised into ‘No Education’, ‘Primary Education’, ‘Secondary Education’ and ‘Higher Education’. Some socio-demographic characteristics of these women were included in the study to access their association with intention of the women to circumcise their daughters in the future. These are age, wealth, religion, region, residence, healthcare decision making, number of daughters already circumcised, frequency of reading newspaper, frequency of listening to radio and frequency of watching television (TV). Following the hierarchical structure of the datasets, all the variables were categorised into individual (education, age, wealth, religion, healthcare decision making, number of daughters, number of daughters circumcised, frequency of reading newspaper, frequency of listening to radio, frequency of watching television) and contextual (family head, region and residence) level factors. To ensure clarity, two of the variables were recoded. These are religion-recoded as ‘Christian = 0’, ‘Islam = 1’, ‘Other = 2’ and number of daughters circumcised recoded as ‘No = 0’, ‘1 = 1’, ‘2 = 2’, ‘3 or more=3.’

### Data analysis

We carried out descriptive analysis as well as two-level multilevel analysis. At the descriptive level, we calculated women’s education and the proportion of women who intended to circumcise their daughters in the future. Chi-square test was conducted in order to determine the socio-demographic variables that significantly relate with intention to circumcise daughter in the future. Due to the clustering and hierarchical nature of the datasets, these analyses were followed by the multilevel analysis conducted with the MLwinN command version 3.05 [39]. Prior to modelling, the dataset was ordered to account for the clustering nature of the survey. A total of four models were constructed with the first one being an empty model in order to ascertain the variance in FGM/C intention at the contextual level (model 1). The second model comprised fixed effects at the individual level (model 2) whereas model three accounted for fixed effects at the contextual level (model 3). In the final model (model 4), fixed effects at the individual and contextual level were fitted. Results of the fixed effects were presented as odds ratios (ORs) and adjusted odds ratios (aOR) together with their corresponding credible intervals (95% Crl). Credible interval was based on the Bayesian statistical approach whereby probability allocation for association measurements are derived whilst 95% credible intervals (95% Crls) are used for summarisation. The 95% CrI implies the possibility of the parameter assuming a value in the specified range [40].

Variance partition coefficient (VPC), also referred to as intraclass correlation coefficient (ICC), and median odds ratio (MOR) were reported for the random effects [41, 42]. The VPC assesses the magnitude of variance in the likelihood of FGM/C intention that is explained by or attributable to contextual factors. The MOR, on the order hand, accounts for the contextual variance in terms of odds ratio and calculates the propensity of FGM/C intention that is explained by contextual factors. Multicollinearity was assessed with the variance inflation factor before the models were developed [43]. Sample weight was applied and the entire analysis was executed with Stata version 13.0.

### Ethics approval

Ethical approval was granted by the Ministry of Health and Sanitation of Sierra Leone as well as the ethics committee of the DHS Program. During the survey, informed consent was sought from all participants. We applied for and were granted access to the dataset by the Measure DHS Program. The dataset is freely available through https://dhsprogram.com/data/available-datasets.cfm.

## Results

### Descriptive results

In Table 1, we presented the socio-demographic characteristics of the women. The same table presents women’s FGM/C intention for daughters in the future by education and socio-demographic characteristics. We realised that at least seven out of ten women (74.8%) had no formal education and only 1.4% had higher education. Nearly all the women without formal education (93.3%) intended to circumcise their daughters whereas 32.4% of those with higher education intended to do the same. Women aged 25–29 (22.8%) dominated whilst only 8.2% were aged 45–49. FGM/C intention for daughter was high among those aged 40–44 and 30–34 as 90.4 and 90.2% respectively revealed their intention to subject their daughters to FGM/C in the future. The proportion of poorest women (22.7%) exceeded women in all other wealth categories especially when compared with the richest (15.2%). A greater section of the poorest women reported FGM/C intention (94.9%) and this did not vary much from women in other wealth quintiles.

**Table 1:** Summary of socio-demographic characteristics of sample (*N* = 6543)

Variable	Weighted Frequency (n)	Percentage (%)	Intends to circumcise daughter in future		X^**2**^, ***p***-value
			Yes (%)	No (%)	
***Individual level***					
**Education**					618.048, *p* < 0.001
No Education	4895	74.8	93.3	6.7	
Primary	845	12.9	88.6	11.4	
Secondary	712	10.9	74.2	25.8	
Higher	91	1.4	32.4	67.7	
**Age**					
15–19	203	3.1	87.6	12.4	5.107, 0.530
20–24	815	12.5	89.8	10.2	
25–29	1490	22.8	90.1	9.9	
30–34	1443	22.0	90.2	9.8	
35–39	1367	20.9	89.0	11.0	
40–44	687	10.5	90.4	9.6	
45–49	538	8.2	87.3	12.7	
**Wealth**					
Poorest	1488	22.7	94.9	5.2	460.818, p < 0.001
Poorer	1426	21.8	93.1	6.9	
Middle	1415	21.6	93.2	6.8	
Richer	1221	18.7	90.5	9.5	
Richest	993	15.2	70.7	29.3	
**Religion**					322.448, p < 0.001
Christianity	1138	17.4	75.3	24.7	
Islam	5390	82.4	92.8	7.2	
Other	15	0.2	86.7	13.3	
**Healthcare decision**					19.549, p < 0.001
Alone	539	8.2	89.8	10.2	
With partner	2993	45.7	87.9	12.1	
Partner	2982	45.5	91.3	8.7	
Other	29	0.4	83.3	16.7	
**Number of daughters**					54.180, p < 0.001
0	854	13.1	85.8	14.2	
1	3132	47.9	87.8	12.2	
2	1746	26.7	92.6	7.4	
3	625	9.6	93.9	6.1	
4 or more	186	2.9	92.7	7.3	
**Daughters Circumcised**					115.497, p < 0.001
None	5129	78.4	87.5	12.5	
1	821	12.5	97.2	2.8	
2	410	6.3	97.6	2.4	
3 or More	183	2.8	98.9	1.1	
**Frequency of reading Newspaper**					411.951, p < 0.001
Not at all	6263	95.7	91.3	8.7	
Less than once a week	103	1.6	59.8	40.2	
At least once a week	177	2.7	52.3	47.7	
**Frequency of listening to Radio**					60.484, p < 0.001
Not at all	2813	43.0	92.2	7.8	
Less than once a week	1428	21.8	90.7	9.3	
At least once a week	2302	35.2	85.7	14.3	
**Frequency of watching TV**					296.693, p < 0.001
Not at all	5732	87.6	91.9	8.1	
Less than once a week	292	4.5	83.7	16.3	
At least once a week	519	7.9	68.5	31.5	
***Contextual level***					
**Family head**					9.217, *p* < 0.01
Male	5176	79.1	90.2	9.8	
Female	1366	20.9	87.5	12.5	
**Region**					391.544, p < 0.001
Eastern	1674	25.6	91.0	9.0	
Northern	2515	38.4	93.6	6.4	
Southern	1529	23.4	91.2	8.8	
Western	825	12.6	68.8	31.2	
**Residence**					248.236, p < 0.001
Rural	1661	25.4	80.6	19.4	
Urban	4882	74.6	93.5	6.5	

*Source* 2013 Sierra Leone Demographic and Health Survey

A statistically significant difference existed between Muslims (92.8%) and Christians (75.3%) with FGM/C intention. A significant proportion of them jointly decided their healthcare with their partners (45.7%), however, 91.3% of those whose partners decided their healthcare had FGM/C intention. Nearly half of the women had one daughter (47.9%), whilst 92.7% of those with four or more children intended to cut their daughters. Among women who did not have daughters, 14.2% revealed FGM/C intention. Most of them had not circumcised their daughters at the time of the survey (78.4%).

Almost all the women who had already circumcised three or more daughters revealed their intention of circumcising other daughters in the future (98.9%). Most of the women were not reading newspaper at all (95.7%), not listening to radio at all (43.0%) and not watching television at all (87.6%). Almost all the women who were not reading newspaper at all (91.3%), not listening to radio at all (92.2%) and not watching television at all (91.9%) reported FGM/C intention. Nine out of ten women in male-headed households intended to circumcise their daughters in the future (90.2%). A significant proportion of them were residents of the Northern region (38.4%) and 93.6% of women in the same region had FGM/C intention in the future. We realised that 74.6% were residing in rural locations and 93.5% of them reported FGM/C intention as illustrated in Table 1.

### Individual and contextual factors associated with FGM/C intention for daughter in the future

As shown in Table 2, women who had no formal education were more than 4 times likely to report intention of subjecting their daughters to FGM/C in the future compared to those with higher education [aOR = 4.3, CrI = 2.4–8.0]. Compared with women aged 45–49, those aged 20–24 [aOR = 2.3, CrI = 1.5–3.4] were more probable to indicate that they intended to circumcise their daughters in the future. Our findings showed that poorest women were more likely to report FGM/C intention for their daughters in the future compared with the richest [aOR = 2.1, CrI = 1.3–3.2].

**Table 2:** Individual and contextual factors associated with FGM/C intention for daughter in the future

	Model 1		Model 2		Model 3		Model 4	
	Empty Model		OR	[95% Crl]	OR	[95% Crl]	aOR	[95% Crl]
**Fixed-effects**								
***Individual level***								
**Education**								
No Education			3.9***	[2.1–7.0]			4.3***	[2.4–8.0]
Primary			2.9***	[1.6–5.3]			3.3***	[1.8–6.2]
Secondary			2.0**	[1.1–3.3]			2.2**	[1.2–3.8]
Higher			1	[1]			1	[1]
**Age**								
15–19			1.9*	[1.1–3.3]			1.8*	[1.1–3.2]
20–24			2.4***	[1.6–3.6]			2.3***	[1.5–3.4]
25–29			2.2***	[1.5–3.3]			2.2***	[1.5–3.2]
30–34			1.9**	[1.3–2.8]			1.8**	[1.3–2.7]
35–39			1.6**	[1.1–2.4]			1.6*	[1.1–2.3]
40–44			1.7*	[1.1–2.6]			1.6*	[1.1–2.5]
45–49			1	[1]			1	[1]
**Wealth**								
Poorest			3.3***	[2.3–4.7]			2.1**	[1.3–3.2]
Poorer			2.3***	[1.6–3.2]			1.4	[0.9–2.2]
Middle			2.6***	[1.9–3.6]			1.7**	[1.1–2.5]
Richer			1.9***	[1.4–2.6]			1.5*	[1.1–2.0]
Richest			1	[1]			1	[1]
**Religion**								
Christianity			0.9	[0.2–4.4]			1.0	[0.2–4.7]
Islam			2.8	[0.6–12.7]			2.9	[0.6–13.7]
Other			1	[1]			1	[1]
**Healthcare decision**								
Alone			1.7	[0.6–4.9]				
With partner			1.6	[0.6–4.6]			1.7	[0.6–5.3]
Partner			1.9	[0.7–5.5]			1.7	[0.6–4.8]
Other			1	[1]			1	[1]
**Number of daughters**								
0			0.97	[0.51–1.86]			0.99	[0.52–1.91]
1			1.25	[0.67–2.33]			1.27	[0.68–2.37]
2			1.80	[0.95–3.40]			1.82	[0.67–2.37]
3			1.59	[0.79–3.17]			1.62	[0.81–3.26]
4 or more								
**Daughters Circumcised**								
None			0.1***	[0.0–0.3]			0.1**	[0.0–0.3]
1			0.4	[0.1–1.6]			0.4	[0.1–1.7]
2			0.4	[0.1–2.1]			0.5	[0.1–2.2]
3 or More			1	[1]			1	[1]
**Frequency of reading Newspaper**								
Not at all			1.6*	[1.1–2.5]			1.6	[0.9–2.5]
Less than once a week			0.9	[0.5–1.5]			0.8	[0.5–1.5]
At least once a week			1	[1]			1	[1]
**Frequency of listening to Radio**								
Not at all			0.9	[0.8–1.2]			0.9	[0.8–1.2]
Less than once a week			1.3	[0.9–1.6]			1.3	[0.9–1.7]
At least once a week			1	[1]			1	[1]
**Frequency of watching TV**								
Not at all			1.4*	[1.1–1.9]			1.2	[0.9–1.7]
Less than once a week			1.2	[0.7–1.8]			1.1	[0.72–1.7]
At least once a week			1	[1]			1	[1]
***Contextual level***								
**Family head**								
Male					1.0	[0.8–1.3]	1.0	[0.8–1.3]
Female					1	[1]	1	[1]
**Region**								
Eastern					3.4***	[2.3–4.9]	2.1***	[1.4–3.1]
Northern					4.3***	[2.3–6.2]	2.2***	[1.5–3.2]
Southern					3.1***	[2.1–4.5]	2.1***	[1.4–3.1]
Western					1	[1]	1	[1]
**Residence**								
Rural					0.4***	[0.3–0.6]	0.8	[0.6–1.1]
Urban					1	[1]	1	[1]
**Random-effects**								
**Contextual level**								
Variance (95% CrI)	1.4	[1.11–1.7]	0.6	[0.4–0.7]	0.7	[0.5–0.9]	0.51	[0.3–0.7]
VPC %(95% Crl)	33.4	[25.4–39.8]	20.7	[13.2–25.9]	23.5	[15.4–28.3]	19.4	[9.1–27.7]
MOR (95% CrI)	3.1	[2.7–3.4]	2.0	[1.8–2.3]	2.2	[2.0–2.5]	2.0	[1.7–2.2]
Explained variation (%)	1	[1]	60.4	[55.4–64.0]	49.6	[46.4–55.0]	63.3	[57.8–71.2]
**Sample Size**								
Contextual level		435	435		435		435	
Individual level		6543	6543		6543		6543	

*aOR* adjusted Odds Ratio, *OR* Odds Ratio, *CrI* Credible Interval, *VPC* Variance Partition Coefficient;

*MOR* Median Odds Ratio; 1 = reference; *p < 0.05, **p < 0.01, ***p < 0.001]

*Source* 2013 Sierra Leone Demographic and Health Survey

However, women who had none of their daughters circumcised were less likely to report FGM/C intention in the future compared to those who had three or more daughters circumcised [aOR = 0.1, CrI = 0.0–0.3]. Intention to circumcise daughter in the future was two times likely among women in the Northern [aOR = 2.2, CrI = 1.5–3.2], Eastern [aOR = 2.1, CrI = 1.4–3.1] and Southern [aOR = 2.1, CrI = 1.4–3.1] regions compared with those in the Western region.

All models revealed remarkable variation in FGM/C intention with respect to contextual factors. The empty model revealed VPC of 33.4%, indicating that the variance in the likelihood of FGM/C intention is substantially attributable to contextual factors. The results for the MOR reflected same with 3.1% [2.7–3.4]. From the final model (model 4), when a woman relocates to a different contextual setting that has a higher probability of FGM/C intention, she stands 2.0% [1.7–2.2] increased chance of FGM/C intention for daughter. Similarly, the final model revealed that contextual factors account for 63.3% of FGM/C intention for daughters. The variance inflation factor (VIF) test revealed that the independent variables were not highly correlated (mean = 1.31, highest = 1.81, least = 1.06).

## Discussion

In this paper, we investigated whether educated women intend to circumcise their daughters in Sierra Leone. This study revealed that women who had no formal education were more than 4 times likely to report intention of subjecting their daughters to FGM/C in the future compared to those with higher education. This result is consistent with community-based study on the change of practice of FGM/C in a Sudanese village where it was demonstrated that the level of education of the woman plays an important role in her decision or attitude towards the practice [44].

This finding is also consistent with the report by Asekun-Olarinmoye and Amusan [45] from their intervention study using a multistage sampling technique in a rural Nigerian community that reported that participants with no formal education were more likely to accept and engage in FGM/C. Formal education exposes people to harmful health practises, risky behaviours and human rights and as such could explain why participants with some level of formal education were not willing to subject their daughters to FGM/C. However, this finding does not support the findings of Allam et al. [46] from their cross sectional study involving university students in Egypt on FGM/C which reports that a considerable amount of educated populace in Egypt including university students, doctors and midwives were ignorant about FGM/C. It is therefore difficult in some situations to assume that formal education equates exposure to issues surrounding FGM/C.

It was found that poorer women were more likely to intend to expose their daughters to FGM/C than those who were rich. This finding is consistent with a Sudanese study which reported high prevalence of FGM/C among the poor [47]. Poverty limits ones access to education and as such could be a major hindrance for such women to be either exposed to the harmful effects of FGM/C or to learn about it. Poverty sometimes puts enormous pressure on daughters’ marriageability, since their sustenance may depend on good marriage. If men embrace such practice and prefer to marry females who have been circumcised, women may feel obliged to circumcise their daughters. Consequently, educating men and boys about the demerits of FGM/C, in addition to females, can expedite the abandonment process of FGM/C because men have a significant role to play [48].

Region and residence of the respondents also played an important role. A significant proportion of women who were residents of rural settings intended to circumcise their daughters in the future. This is consistent with the findings of Alo and Gbadebo [49] from their study on intergenerational attitude changes regarding FGM/C in Nigeria that showed that females in a rural setting were more likely to support FGM/C compared to those living in urbanised communities. This is expected considering the fact that most women who find themselves in the rural areas are accustomed to tradition, have low levels of education and have little or no access to information and media as compared to those who live in urban areas. With more development programs in urban areas, it is likely for urban residents to have exposure to or receive health education and information on the harmful effects of the practice for informed decision making on whether or not to allow their daughters to be circumcised.

Comparing women aged 45–49 to those aged 15–19 and those within 20–24 age bracket, the 15–19 and 20–24 age brackets were thrice more probable to indicate that they intend to circumcise their daughters in the future. This is an indication that the younger women were more likely to circumcise their daughters in the future compared with their older counterparts. The older counterparts could be exposed to more education on the effects of FGM/C and as such can make more informed decisions and choices for their daughters as compared with their younger counterparts. This is consistent with the account of Rahlenbeck and Mekonnen [50]. They indicated that lower age categories were associated with increased odds of FGM/C among women. This finding is however in variance with the findings from a study conducted by Alo and Gbadebo [51] in South Western Nigeria where they noted that older respondents were more likely to accept FGM/C as compared to their younger respondents. The assumption is that younger respondents were more likely to be educated which will increase their chances of engaging with FGM/C education and their associated harmful effects, thereby influencing their decisions.

Women who do not read newspapers at all were more likely to indicate that they intend to circumcise their daughters in the future compared with women who read newspapers at least once a day. This can be attributed to the fact that women who read newspapers are more exposed and informed about issues surrounding human rights and harmful health practises. They are more likely to know and understand negative effects of FGM/C. Malhotra et al. [52] mentioned that women who read are more likely to weigh the benefits over the risks before making decisions about their health. A clear understanding of the benefits of not circumcising is likely to be attractive to women. In addition, when women are able to make autonomous decisions, they are able to take actions that will favour them [53].

The study revealed that contextual factors account for women’s FGM/C intention in Sierra Leone. This implies that FGM/C intention transcends beyond the individual or personal level factors. This finding is consistent with reports by the UNFPA and other Sierra Leone based FGM/C studies [6, 54, 55]. As such, there is the need for the government and anti-FGM/C institutions in Sierra Leone to focus on wider social norms. This is needful because contextual factors may greatly condition the impact of maternal education on women’s FGM/C intention [56].

Due to the cross-sectional study design, our results do not lend itself to causal inference. There is also the possibility of under or over reporting based on the women’s evaluation of how society expects them to react to FGM/C. The data supporting this study emerged from a survey conducted in 2013, though remains the most current of the DHS in Sierra Leone as of now. As a result, this may not reflect the current phenomenon, however, it presents an accurate findings of nationwide coverage which can direct FGM/C policies. This study involves a nationally representative sample which can be generalised not only to Sierra Leone but other low and middle income countries in Africa.

## Conclusion

 Women with low levels of education are more probable to circumcise their daughters in the future. Poor women also have high FGM/C inclination. Formal education is essential and critical to stopping FGM/C in Sierra Leone. Efforts to reduce FGM/C among women requires a strategy that will focus attention on strengthening and enforcing the existing laws on FGM/C as well as formulating policies that will reduce poverty among rural women especially women in the rural Sierra Leone where the practice is endemic. Pro female child education policies must also be introduced to achieve the desired set targets.

## Data Availability

The datasets supporting the conclusions of this article are available in the Measure DHS repository, https://dhsprogram.com/data/available-datasets.cfm.

## Abbreviations

AOR: Adjusted Odds Ratio
CI: Confidence Interval
CrI: Credible Interval
EA: Enumeration Area
FGM/C: Female genital mutilation/cutting
FC: Female circumcision
MOR: Median Odds Ratio
MoU: Memorandum of Understanding
OR: Odds Ratio
PSU: Primary Sampling Units
SLDHS: Sierra Leone Demographic and Health Survey
SSL: Statistics Sierra Leone
TV: Television
VPC: Variance Partition Coefficient
WHO: World Health Organisation
